# Optimising the Hydraulic Retention Time in a Pilot-Scale Microbial Electrolysis Cell to Achieve High Volumetric Treatment Rates Using Concentrated Domestic Wastewater

**DOI:** 10.3390/molecules25122945

**Published:** 2020-06-26

**Authors:** Daniel D. Leicester, Jaime M. Amezaga, Andrew Moore, Elizabeth S. Heidrich

**Affiliations:** 1 School of Engineering, Newcastle University, Newcastle-upon-Tyne NE1 7RU, UK; d.leicester@ncl.ac.uk (D.D.L.); jaime.amezaga@ncl.ac.uk (J.M.A.); 2 Northumbrian Water Limited, Northmbria House, Abbey Road, Durham DH1 5FJ, UK; andrew.moore@nwl.co.uk

**Keywords:** bioelectrochemical systems, microbial electrolysis cells, pilot-scale, return sludge liquor, volumetric treatment rate, hydraulic retention time

## Abstract

Bioelectrochemical systems (BES) have the potential to deliver energy-neutral wastewater treatment. Pilot-scale tests have proven that they can operate at low temperatures with real wastewaters. However, volumetric treatment rates (VTRs) have been low, reducing the ability for this technology to compete with activated sludge (AS). This paper describes a pilot-scale microbial electrolysis cell (MEC) operated in continuous flow for 6 months. The reactor was fed return sludge liquor, the concentrated filtrate of anaerobic digestion sludge that has a high chemical oxygen demand (COD). The use of a wastewater with increased soluble organics, along with optimisation of the hydraulic retention time (HRT), resulted in the highest VTR achieved by a pilot-scale MEC treating real wastewater. Peak HRT was 0.5-days, resulting in an average VTR of 3.82 kgCOD/m^3^∙day and a 55% COD removal efficiency. Finally, using the data obtained, a direct analysis of the potential savings from the reduced loading on AS was then made. Theoretical calculation of the required tank size, with the estimated costs and savings, indicates that the use of an MEC as a return sludge liquor pre-treatment technique could result in an industrially viable system.

## 1. Introduction

Bioelectrochemical systems (BESs) are a wastewater treatment technology that use anaerobic and electrochemically active microorganisms (EAMs) within the wastewater to break down pollutants and recover energy. Typically, they consist of two chambers, one containing an anode and the other a cathode. Most of the research for BESs has been at a laboratory-based scale of millilitre to litre volumes, and scaling up these reactors still remains a challenge [1,2]. This is due to a number of reasons: pilot reactors are expensive; they are difficult to engineer; scale-up shows decreased performance; and data from a pilot-scale reactor is often limited and rarely in duplicate. Therefore, only a small number of pilot-scale BESs have been built, as reviewed by Rousseau et al. [3] and Gajda et al. [4]. All use a range of configurations, operate under different conditions and use different substrates. Standardising and comparing the results from these studies is difficult, as even the phrase pilot scale can be ambiguous. To call a reactor ‘pilot-scale’, Wang et al. [5] determined that it must fulfil certain criteria: the reactors must be in operation for greater than two months, be continuously flowing, be run using real wastewater, and have 0.1 to 5% of the practical flow of the wastewater facility. The majority of published pilot or semi-pilot BESs do not fit these criteria.

The most common configurations of BESs are microbial fuel cells (MFCs) and microbial electrolysis cells (MECs) [6]. As MECs are able to recover valuable hydrogen gas rather than electricity, it has been suggested that scaling up an MEC is more economically viable than scaling up an MFC [7,8]. MFCs produce current that is simple to use, but do so only at a very low level. They also require oxygen at the cathode, and therefore need constant aeration or the use of an air-cathode. Constant aeration requires extra energy and the largest air-cathode to date is only 0.62 m^2^ [8]. The hydraulic pressure exerted on air-cathodes poses not only structural difficulty, but also increases charge and diffusion transfer resistance, as well as suppression of bacteria in the biofilm [9]. An MEC has the advantage that both chambers are anaerobic with no oxygen required, meaning they may be more able to fit into the infrastructure of large-scale treatment works.

Currently, six MEC studies have been published as pilot-scale [1,2,10,11,12,13], one described as technical-scale [14], two as scale-up [15,16], two as semi-pilot [17,18] and one as a mini-pilot [19]. The first and still largest to date was a 1000 L MEC treating winery wastewater [1], which successfully removed ~70% of the influent chemical oxygen demand (COD). Unfortunately, due to the single chamber design and heating of the reactor, methane was produced rather than hydrogen. However, it was still energy positive (+14.3 kJ/gCOD). Since then, there have been a number of ‘proof of concept’ designs, which have successfully produced hydrogen while also treating wastewater. The most successful reactors include Carlotta-Jones et al. [19] in terms of hydrogen production (0.066 m^3^/m^3^·day), Isabel San-Martin et al. [11] in terms of COD removal efficiency (84%) and Cotterill et al. [2] in terms of COD volumetric treatment rates (VTRs) (1.06 kgCOD/m^3^∙day). However, none of these reactors achieved *both* discharge standards and net energy recovery from hydrogen capture, and VTRs were far lower than can be achieved with activated sludge (AS). The technology is still not ready for industry, and performance at a pilot-scale falls short of the performance needed to make it competitive with current technologies.

Application into the commercial sector is an enormous challenge. The water industry is typically conservative and risk adverse, being driven by the need to comply with regulation more than to innovate. BESs are unlikely to leap from academic research to full-scale water treatment. If this technology is to progress, it will be important to find places where there is a need or economic drive that cannot currently be met by other technologies. This could be in developing countries where there is little existing safe sanitation [20], or it could be treating high concentration wastes which are too liquid to feed a standard anaerobic digestion system [21]. Developing a minimal viable product that is usable and can add value could allow entry of BES technology into the water industry, enabling further commercial development and cost reduction to take place.

Most of the pilot-scale work to date has used domestic wastewater, before or after primary sedimentation [2,10,11,13]. Although the goal would be to eventually replace the energetically expensive AS [7], the change required to achieve this would be a huge undertaking and the risks of failure prohibitively high. Alternative waste streams within the treatment process could provide a safer option, and introduce this new technology into the commercial sector. A recent drive to generate renewable energy and improve the efficiency of the treatment works has seen anaerobic digestion added to many large treatment sites in the UK and elsewhere [22]. At these treatment sites, following the AS treatment process, waste sludge is dewatered and then anaerobically digested. During this process, energy is recovered in the form of biogas while the sludge is further treated. The effluent from this process is then dewatered again, with the bio-solids safely and beneficially re-cycled to land. The combined liquid fraction from these dewatering steps returns to the top of the treatment works and passes back through the AS process in an internal loop. This waste stream is one of the most energetically resource-laden sections of the wastewater treatment plant, and currently there is no economically attractive solution [23]. This return sludge liquor (RSL) is much higher strength than raw sewage, i.e. it contains more organic matter. Importantly, a high proportion of this organic matter is soluble and therefore may be more biologically available. The organic content, represented by the COD values, ranges from 1500–6000 mg/L (or 24.15–96.6 kJ/L [24]) in RSL, whereas low to medium strength raw wastewaters have COD values of 300–700 mg/L [25] (or 4.83–11.27 kJ/L).

At a large treatment plant such as Howdon Wastewater Treatment Works in the North East of England (Northumbrian Water Ltd), this RSL may have a flow rate of 100 L/s and an average COD of 2500 mg/L. The energetic treatment cost for AS is predicted to be between 1.08 and 2.1 kJ/gCOD [26], or 2.52 and 7.2 kJ/gCOD [27]. Therefore, using an ambitious but realistic energetic treatment cost of 2 kJ/gCOD, with an average energy cost (for business rates) of £0.13 /kWh or £3.6 × 10^−5^ /kJ [28,29], treating this RSL in the AS process could cost the treatment plant around £1500 a day. As BESs have shown increased performance when treating a substrate with a high COD content [30,31], this waste stream could be an ideal location for a pilot-scale BES, where it may be possible to get economically viable treatment rates. In addition, as the RSL simply returns to the top of the treatment process rather than being discharged, there is no demand to meet effluent standards. Any reduction in COD and energy recovery will be beneficial from the reduced loading on the AS process.

BES have large capital costs [32], therefore it is important that the reactors perform to their highest potential. One way to achieve this could be to optimise operational conditions, such as the hydraulic retention time (HRT), which would then have consequential effects on performance parameters, such as the VTRs. The HRT of these systems describes the length of time the wastewater remains in the reactor. It is a function of the volume of the tank and the flow rate, which determines the organic loading rate (OLR), and the contact time between the wastewater and the biofilm. Faster flow (or lower HRT) increases the OLR as more food is delivered into the tank. Increasing the OLRs by using high strength wastewaters has been shown to improve the performance of BES in terms of the VTRs [31]. As the HRT will affect both the OLR and the subsequent pollutant removal, as well as electron transfer and hydrogen/electricity recovery, the HRT is a critical operational parameter for MECs [33]. If the HRT is not optimised, pollutant diffusion may be limited, and the reactors could underperform. Furthermore, tanks may be built to the wrong size, adding to their cost.

**Figure 1:**
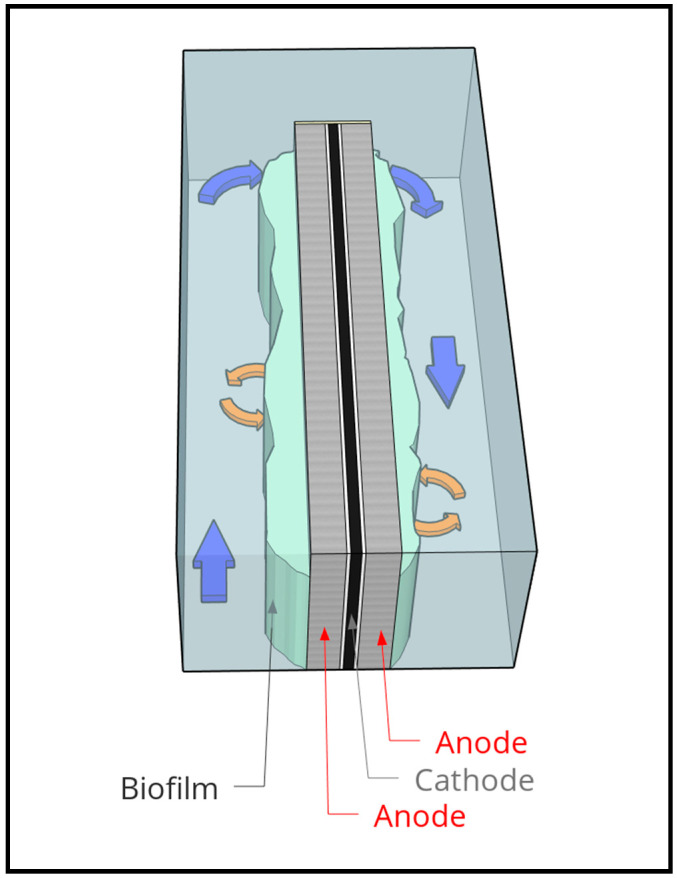
A simple diagram to represent wastewater flowing around the cassette style electrodes.

Despite the importance of optimising HRT, this has not been done in most of the previous pilot scale studies. Many pilot reactors use different architectures. However, three have been based on the cassette design outlined by Heidrich et al. [10]. This is where wastewater flows around rectangular cassette style electrodes with two external anodes and an internal cathode (Figure 1). In these studies, the reactors were run using different HRTs and supplied different voltages. Cotterill et al. [2] used a HRT of 5 h and inputted 0.9 V; Heidrich et al. [10] used a HRT of 1 day and inputted 1.2 V, and Baeza et al. [13] ended up using a 2-day HRT with 1.5 V. As these were proof of concept reactors, conditions to maximise the reactor performance were not optimised by Heidrich et al. [10] or Cotterill et al. [2], with no explanation as to why these conditions were chosen. Baeza et al. [13] switched from a 1-day to a 2-day HRT to improve COD removal, successfully increasing it from 6% to 25%. At the end of the experiment, they determined that a 10-day retention time would be needed to reach the desired ~75% COD removal; however, full optimisation over a range of HRTs was not measured. Although the reactors were of the same design, the different operational conditions used makes it impossible to compare results between them.

A HRT of 1 day has been most commonly used [1,10,11,34,35], while pilot reactors operating in batch have gone as long as 10 days [13]. The most thorough attempt to measure how HRT affects reactor performance was by Gill-Carrera et al. [17], measuring HRTs of 25, 23, 11, 7 and 4 h in a two-chamber semi-pilot MEC, operated within a lab and kept at room temperature. Focusing on the first chamber, a longer HRT enabled greater coulombic efficiency (CE) (efficiency of anodic electron transfer), peaking at 94.3%. However, a shorter HRT showed increased cathodic conversion efficiency (CCE) (efficiency of electron recovery as hydrogen gas). The reactors used low strength wastewater as the influent substrate (<112 mg/L), and therefore were subject to OLRs well below typical AS loading rates, leading to low VTRs. Similarly, Gill-Carrera et al. [18] measured three HRTs and two voltages in a semi-pilot tubular MEC. It was determined that 4 h and 1 V were the optimum conditions in terms of hydrogen recovery and 10 h and 1 V in terms of pollutant removal. Using a pilot-scale multi-anode/cathode MFC, Jiang et al. [36] measured the performance at three HRTs, determining that COD removal efficiency increased from 66% to 80% from a 5 to 20 h HRT.

This paper describes a pilot-scale MEC operated using high concentration RSL at Northumbrian Water Ltd.’s Howdon Wastewater Treatment Plant, June 2019–February 2020. It aims to improve the effectiveness of the anode side of the cassette style electrodes by determining the optimal HRT with respect to COD removal efficiencies, VTRs and current densities. Using the data obtained and existing costing models, a direct analysis of the potential savings from the reduced loading on AS is then made.

## 2. Results

### 2.1. Start-Up and Operation

Acclimatisation was rapid. Current was observed in two of the electrodes after two days, with seven more starting after five days. Gas was produced after 16 days. The final electrode produced a current after week six following wiring replacement (Electrode 8). Although there was variability between electrodes, average current density increased for the first 29 days before stabilising around 1.17 A/m^2^. Gas production increased steadily throughout the first four weeks, reaching 0.007 m^3^/m^3^∙day. Following the 38 days of batch cycles, the reactor was switched to continuous flow. At the start, the reactor was run using a 0.25-day HRT. Due to an event on site termed ‘core settling’, high strength thick sludge was pumped into the reactor on day 57, causing it to flood and overflow. This reduced gas production and seemed to alter current densities for each electrode. In some cases, this was a reduction (e.g., Cassette 3), whereas in other cases it increased (e.g., Cassette 4). The HRT was then switched to 0.5 days for a further 2 weeks. Following this, the reactor was then deemed stable enough, and the different HRTs were tested over the following 6 months.

During 90 days of stable performance under continuous flow, gas production continued at all HRTs excluding the 0.015-day HRT. At day 134, another ‘core settling’ event occurred and completely flooded the cathode compartment of the electrodes. As a result, gas production stopped, most likely due to microbial contamination; however, current generation remained stable. As this is an immediate indication of the microbial substrate unitisation (hydrogen production being a secondary chemical process occurring at the cathode), the HRT optimisation experiment continued. Full wastewater components were measured prior to the experiment and can be found in Table 1. Spot samples of pH were taken throughout the experimental period and compared in different sections of the reactor; pH remained stable throughout the operation.

### 2.2. HRT Optimisation

The eight HRTs varied between 0.015 days and 18 days. For each HRT, current densities from all 10 electrodes were recorded and the average used for the whole reactor. For HRTs under 6 days, the reactor was operated at that HRT for a minimum of 3 weeks, producing at least triplicate data. The longer cycles were operated in batch, producing triplicate 6-day results and duplicate 18-day HRT results. All the replicated HRTs were measured in a randomized order to reduce the confounding effect of change in the biofilm over time. Influent COD was measured at the start of each cycle and the effluent at the end. The relationships between the performance indicators and HRT can be seen in Table 2.

**Figure 2:**
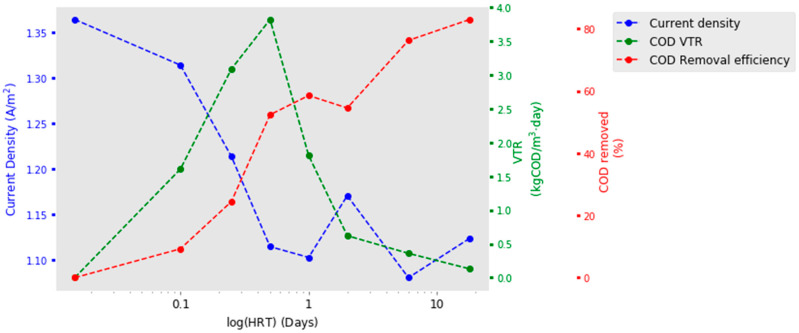
Current density, chemical oxygen demand (COD) removal efficiency and COD volumetric treatment rates (VTRs) with comparison to Log (HRT).

The VTR shows a clear pattern with HRT, the optimum being 0.5 days, as shown in Table 2 and Figure 2. The fastest HRT of 0.015-days achieved no measureable COD removal with a VTR of 0-kgCOD/m^3^∙day. Increasing the HRT increased VTRs until a 0.5-day HRT, where it peaked at 3.82 kgCOD/m^3^∙day. The rise shows a logarithmic increase (r^2^ = 0.99) and, following this peak, VTRs exponentially decrease (r^2^ = −0.862). The hydrogen gas production appears as though it may have followed a similar trend, peaking at a HRT of 0.25-days; however, the incomplete data set makes this difficult to verify. The removal efficiency, i.e., the percentage of COD removed as the wastewater passes through the tank, predictably increases with HRT. However, it does not do so at a steady rate (Figure 2). Between HRTs of 0.015 and 0.5 days, removal efficiency sharply rises. Following this, for HRTs of 0.5, 1 and 2 days, it plateaus and the removal efficiency fluctuates between 52% and 58%. For the longest HRTs measured (6 and 18 days), a further increase is seen, with 18 days seeing the highest COD removal with 84%. Fitting a linear regression to log (HRT) and removal efficiency suggests a logarithmic increase as HRT increases (r^2^ = 0.965). Visually on Figure 2, it can be seen that peak reactor performance for VTR sits at a HRT of 0.5-days. Doubling the 0.5-day HRT to a 1-day HRT halves the VTR from 3.82 to 1.81 kgCOD/m^3^∙day, but only rewards with a 5% increase in overall COD removal.

Current density did not follow the expected optimisation curve, nor did it mirror the VTR data. The highest current density was at the fastest flow where there was no measureable wastewater treatment. Current density then declined with increasing HRT until 0.5 days (Figure 2). With longer HRTs, between a 0.5-day and an 18-day HRT, there was no further decrease and current density fluctuated between 1.08 and 1.17 A/m^2^. Fitting a linear regression to log (HRT) and current density gives an r^2^ value of −0.832 and suggests current density exponentially decreases with increasing HRT. However, although there was a decline in average values over the HRTs, the range observed was very small (0.25 A/m^2^). This is in comparison to the large range in current densities observed between the 10 cassettes (2.15 A/m^2^) (see Supplementary Information, Figure S1). Examining each cassette individually confirms that there was not a clear pattern between the HRT and the current density. Fitting regression lines to these resulted in a range of r^2^ values from 0.142 (Cassette 4) to −0.96 (Cassette 2) (see Supplementary Information, Figure S1). CE was shown to increase with HRTs. However, only at HRTs of greater than 1-day was there any reasonable recovery. No trends were seen with cathodic conversion efficiency (CCE) with respect to HRT.

### 2.3. Energetic Cost

The average VTRs achieved at different HRTs, along with the voltage input and average current densities, have resulted in an energetic removal cost for this reactor at each HRT measured (Table 2). This can be compared to the equivalent cost of AS to achieve the same VTRs, using an energetic treatment cost of 2 kJ/gCOD for AS [26,27]. For HRTs between 0.1 and 1 day, the MEC reactor removed the organics using less energy than would AS. At a HRT of 2 days or greater, this reactor was energetically more expensive than the AS equivalent. The reactor removed no pollutants when subject to a 0.015-day HRT and therefore used more energy than AS.

### 2.4. The Effect of Influent COD Concentrations

In order to calculate the length of the tank that would be needed to remove fully the organics to legal discharge standards, and to understand if the optimal HRT was still relevant with lower strength wastewaters, a further experiment was conducted where the RSL was continually recirculated through the tank. Initially, the RSL flow rate was set to produce a 0.5-day HRT, and therefore was set to 3 L/h. After the first fill of raw RSL, the effluent was piped back to the influent point of the reactor. The flow was continuously recirculated until the COD dropped to 280 mg/L COD. The rate of removal at these low COD values was so slow it was decided not to continue until UK discharge standards, as had been previously proposed (125 mg/L). The COD was measured at the influent and effluent at the same time each day. In the case of the 0.5-day HRT, it was impossible to gain access to the site and sample at regular 12-h intervals, and so measurements were taken every 24 h. The VTRs were calculated based on the two circulations the RSL had achieved. This data was supplemented using the four repeated cycles for the initial HRT experiment, which due to the natural variation in the RSL, had influent CODs ranging between 4320 and 2186 mg/L (Table 3).

**Figure 3:**
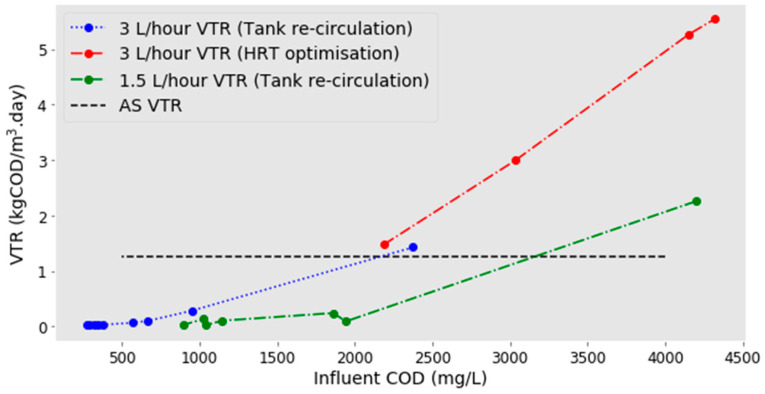
A graph to represent how the influent COD affects the VTR. Data collected during HRT optimisation is shown in red (3 L/h), and data collected from RSL recirculation can be seen in blue (3 L/h) and green (1.5 L/h).

It was possible to combine the two data sets from the replica 0.5-day HRT cycles (Figure 3, red line) and the recirculation data (Figure 3, blue line) as there is reasonably good agreement at the point they meet. It can be seen that the VTR clearly increases as the COD in the influent increases. Between the influent CODs of 4320 and 2186 mg/L from the HRT optimisation, VTRs have a linear increase with increasing COD (Figure 3, red line) (r^2^ = 0.994). At lower influent CODs, VTRs increase exponentially with increasing COD in the influent (Figure 3, blue line) (r^2^ = 0.948) when fitting a linear regression to log (influent COD) and VTRs.

Following this, the same recirculating technique was completed with a slower flow rate. It was hypothesised that as COD was reduced, the lower flow rate would be beneficial and result in higher VTRs due to the increased contact time between the biofilm and the RSL. The reactor was again filled with RSL, and then the effluent was pumped back to the top at 1.5 L/h (the equivalent to a 1-day HRT). A similar pattern was observed, but the VTR was generally lower than at the faster flow, and at CODs of 2000 mg/L and less, VTR was virtually zero. The data shows the hypothesis to be incorrect: the slower flow offers no advantage over the faster one, especially at low COD (Figure 3, green line).

### 2.5. Tank Design

The data collected in Figure 3 can be used to extrapolate the design of the tank. It is seen that even for this small RSL waste stream, and an optimised HRT, the tank would need to be extremely large.

**Figure 4:**
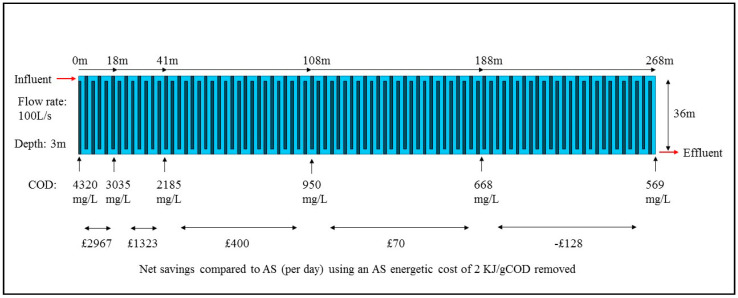
A theoretical tank design required for the actual flow rate found at Howdon Wastewater Treatment Plant (NWL). Values have been calculated based on experimental values from Section 2.4.

When running at the optimum HRT, energetic treatment costs were calculated for each individual influent COD. These can be compared to the energetic treatment costs for AS (which is currently the method used to treat this waste), modelling the difference in energetic costs of a full scale BES reactor. From Figure 4, it shows there is a high energetic cost saving of £2967 per day in the first 18 m. Following this, savings decline and then turn negative, with the effluent only reaching 569 mg/L (higher than discharge standards) after a tank length of 268 m and total size of 28,944 m^3^. Between an influent of 668 mg/L and 569 mg/L, a higher COD concentration than would be found in typical raw domestic wastewater, the MEC was energetically more expensive than AS (>2 kJ/gCOD) (Table 3). 

To become a viable system, the savings to the energetic or running costs would need to be very high to overcome the high capital costs of BESs. A full analysis of the capital costs of this reactor was beyond the scope of this study and is covered elsewhere [32]. However, energetic cost savings in the first part of the tank, removing around 50% of the COD (0–41 m), equate to over £2000 per day, making annual cost savings of £730 K. If the infrastructure lasted over 5 years, it could cost £3.5 million and remain profitable. A recent cost–benefit analysis by Aiken et al. [32] states that in order for MECs to become viable, an increase in OLR and a 90% reduction in anode and current collector costs is needed. The proposed OLR targets of 0.8–1.4 kg COD/m^3^∙day by Aiken et al. [32] were met by this reactor, though anode costs remained high at £285.9/m^2^. Using the model produced by Aiken et al. [32], which was based on a similar pilot design, the capital costs of this reactor would be £61,754 per meter of tank. For the first 18 m alone, the tank would cost £1,144,301, yet with a saving of £2967 per day, due to the high rate of COD removal by the BES, this requires a life span of just over a year (386 days) to break even (if performance seen in this study remained the same). Assuming a 2-year life span of the reactor, the first 41 m of the tank (effluent quality of 2185 mg/L) would be cost effective. This will increase to 108 m (effluent of 950 mg/L) with a 5-year life span. With a 10-year life span, the theoretical tank would be cost effective until an effluent of 668 mg/L was achieved (188 m).

### 2.6. Variability

**Figure 5:**
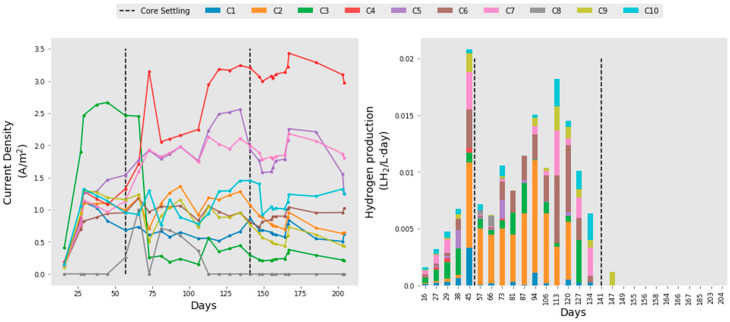
Current density (left) and hydrogen production (right) during start-up, batch mode and continuous flow in each individual electrode. Continuous flow started on day 39.

Within the reactor were 10 identical electrode cassettes, placed in sequence to generate a serpentine flow. The averages from all 10 of these electrode cassettes were taken to compare the whole reactor for the HRT experiments. However, there was extremely high variability between the electrodes in terms of their current density and hydrogen production. During the initial batch mode, all electrodes except Cassette 3 and Cassette 8 exhibited similar current densities. When the reactor was switched to continuous flow, current densities started to vary, and this continued until the end of HRT optimisation (Figure 5). Core settling events also appeared to alter specific electrode current densities, although not in a systematic way. Following core settling, Cassette 3, which previously was producing the highest current at 2.5 A/m^2^, dropped down to 0.25 A/m^2^, where it remained for the duration of the experiment. Comparatively, Cassette 4 increased at the same point, and remained the highest for the duration of the experiment, peaking at 3.4 A/m^2^.

Similarly, this variability was seen in hydrogen production (Figure 5). During batch mode, the majority of the hydrogen gas was generated by Cassette 3. When switched to continuous flow, Cassette 2 generated the most and remained this way until day 113. From day 113, Cassette 6 started to produce similar amounts to Cassette 2 and then from day 127, Cassette 2 ceased hydrogen production. There was no correlation between high current density in certain electrodes and high hydrogen production (r^2^ = −0.296). There also was no apparent trend between performance and positioning within the tank. At day 134, the second ‘core-settling’ event occurred, and hydrogen production stopped in all cassettes.

## 3. Discussion

If MECs could perform to the same level or greater than AS, the switch to a more sustainable wastewater treatment method would be both economically and environmentally beneficial. The performance that needs to be matched is that of COD removal, as the production of current or other products is likely to be a side issue. The VTRs of wastewater treatment systems is vital, as it is a function of both pollutant removal and wastewater flow. It is needed to calculate treatment cost and design treatment tanks. Previous work has highlighted that optimising the HRT of reactors could boost this performance [17,18]; however, this has not been systematically attempted at pilot-scale.

The research presented here shows that the MEC design that has been used in several pilot studies [2,10,13,19,34] can match the treatment rates of AS. However, it can only do so with high concentration wastewaters with a COD of above ~2200 mg/L, or energy content of 35.4 kJ/L [24]. The HRT, or the speed of flow, was found to have a consequential effect on the VTRs. Eight different HRTs were measured in triplicate cycles, and it was found that 0.5 days was the optimal HRT. This equated to a flow of 3 L/h, or 0.4 m/h of flow past the electrode. At this speed, the average VTR was 3.82 kgCOD/m^3^∙day. The optimal HRT (0.5 days) was faster than the HRT that most previous pilot scale experiments have chosen to use, meaning tank sizes would be smaller and costs therefore lower. Typical HRTs of AS are 0.3 days [37], slightly faster than the MEC HRT. However, for anaerobic digesters, where the upstream metabolic processes are putatively the same, these are much longer, between 12 and 30 days [21]. This optimal HRT and subsequent speed of flow seems to be relevant even at low COD concentrations, although here the VTRs are much slower.

It would be expected that the trend in current density would follow the trend of VTRs [38]. In a BES, complex wastes are sequentially broken down by different consortia of bacteria and are eventually funneled into the electrode as electrons. Although it is well documented that there are inefficiencies, dead end paths, and competitive reactions that occur in a BES [39,40,41,42], COD removal should, to some degree, be represented by the current. In this study, this was not the case, which was observed in two distinct ways. Firstly, the optimal HRT with respect to current density was at 0.015-days, where no measureable COD was removed, and secondly, at lower HRTs most of the COD removed did not result in current. We hypothesise that the current recovered at high HRTs was that from the small amounts of the most readily available organics in the waste stream. In the raw RSL, the quantity of acetate measured was 274 mg/L (when the total COD was 4535 mg/L). At 100 L/h flow, this acetate could account for 12.2 coulombs of charge per second, which, distributed over the anodic surface area, would be 15.6 A/m^2^. The current density of 1.36 A/m^2^ at the fastest flow could have been produced by acetate consumption alone, yet as the acetate only makes up around 6% of the total COD, its removal could easily fall below the detectable COD removal values. At lower flows, acetate had to be provided by the breakdown of longer organics, producing a similar but slightly lower current. The results suggest that maintaining an adequate supply of acetate may be a critical factor in current production, as was observed by Fei et al. [39].

With lower flow rates, the disconnect between current production and COD removal was more surprising. At HRTs of between 0.5 and 2 days, over 50% of the COD was removed, yet only 5.6–27.9% of this was transferred to current. Oxygen diffusion into the surface waters is low [34], accounting for less than 1% of the COD removed (calculated using Henry’s Law and an oxygen diffusion coefficient of 769.23 L·atm/mol [43] over the top surface area of the reactor). Sulphate reduction could account for between 3.3 and 7.3% of the COD removed at HRTs of 0.5 and 2 days respectively. This leaves between 63.8% and 90.1% of the COD removed unaccounted for. Some of this may have been converted to biomass, and although neither the build-up of sludge nor the thickness of the biofilm were measured quantitatively, there was no observed increases in these in the reactor. This suggests that much of the COD was anaerobically digested, with the resulting methane released from the open anode section. This is surprising: the reactor was run at ambient UK temperatures (wastewater remained between 10 and 20 degrees [44]), which would normally prohibit anaerobic digestion; it was fed wastewater deemed too liquid to be suitable for the anaerobic digestion process; and it was operated at residence times much lower than typical anaerobic digestion [45]. Intriguingly, this suggests that the BES reactor facilitates high rates of anaerobic COD removal, yet this COD is not being converted into current, or subsequently hydrogen.

Although coulombic efficiencies (CE) were low, indicating a loss of electrons to other reactions, they did increase with increasing HRTs. This suggests that a greater amount of acetate, and then current, was produced the longer the wastewater remained within the reactor. Logically, this should also be seen throughout the reactor’s serpentine flow, with electrodes set at the end being fed an increased amount of predigested wastewater compared to those at the beginning. The exponential decrease seen when comparing the average current density from the whole reactor with HRT should therefore be much stronger in these later cassettes. This was not the case. Cassette 2, 5 and 9 were the only electrodes to show the same significant trend as represented by the total reactor (see supplementary information, Figure S1), with Cassette 2 being the most significant (*p* = 0.0002). Neither current density nor CE appear to be a good indicator of the optimum HRT with respect to wastewater treatment, and are in fact more representative of acetate uptake than organic removal.

The production of current from COD removed was likely to be limited by the development and function of the electrogenic biofilm, which was variable across the reactor. If acetate supply was the main driving force for current production, we would expect to see high current in Cassettes 1 and 2 where the acetate rich wastewater was fed in. We would also expect to see a second area of higher current towards the end of the reactor flow, once more acetate had been produced by the breakdown of longer organic chains. This pattern should be more significant when the flow was low. However, there was no such pattern, in fact it was the cassettes in the mid-section of the reactor which appeared to perform better. High variability between replica cassettes has been observed previously at pilot scale [2,10] and is a significant limitation of this technology. The results suggest that biofilm formation is critical to ongoing performance. In the first 25 days, current production was similar in all cassettes. However, once the reactor changed to continuous flow, this performance separated. Aside from Cassette 3 (which appears to have been harmed in the first core-settling event) this separation was maintained throughout. Devising strategies that can help engineer optimal biofilm development [46], which are workable at larger scales, will be necessary for future developments of this technology.

Such strategies should overcome the performance limitations of low current production from the wastes digested. However, it is also clear that higher efficiency of current conversion into hydrogen production is required to reach energy neutrality. During the hydrogen-producing period, peak production was 0.02 m^3^/m^3^·day, higher than that achieved by Heidrich et al. [10], where hydrogen averaged 0.015 m^3^/m^3^·day, although still only one-third that of Carlotta-Jones et al. [19], which achieved 0.066 m^3^/m^3^·day. In this study, the higher average current densities drew more power, and the energy recovered did not offset the energy added. Events on site also led to contamination of the cathodic chamber, as has been reported in other studies [2], highlighting a potential problem with the reactor design or its operation. If run as an abiotic chamber, a mechanism to routinely sterilise the cathode compartment needs to be developed, such as heat sterilisation, or in situ caustic or peroxide production. It has been reported that hydrogen-scavenging bacteria, such as hydrogenotrophic methanogens, can result in the loss of hydrogen from the cathode chamber [1,2]. This could be the case in this reactor, particularly as contamination was visible during the core settling events. This would result in the production of methane. However, at the point of no hydrogen production, there was no gas produced at all within the cathode gasbags. It is therefore possible that gases diffused back into the anode side and released into the atmosphere. A further problem with hydrogen production is again the variability between electrode cassettes. This variability did not mirror that observed in current production. Cassette 2 was responsible for almost half of all the total hydrogen production, yet its current density was low throughout. With large-scale reactors, current at the anode is not the controlling factor for hydrogen production. It is possible that minor differences in the internal resistance in each cassette caused some of this variability, but further investigation into low hydrogen yields will be paramount for commercialisation.

High removal efficiency, i.e. the percentage of COD removed, is desirable and often a main focus in pilot-scale work. Wastewater treatment relies on the ability to meet discharge standards, which in the UK are 125 mg/L COD, or >75% removal [47]. The removal efficiency in this reactor at a 0.5-day HRT was around 50%. This is lower than several previous pilot studies, all of which use low strength wastewaters [1,11,14,35], but with a longer HRT. This research showed that longer HRTs enabled greater breakdown of organics most likely in both the bulk liquid and in the biofilm, allowing the slower rate limiting steps, such as hydrolysis [48], to have occurred. This subsequently led to an increase of CE, as prolonged exposure to the biofilm enabled greater conversion of complex organics to electrons. The logarithmic increase between increasing removal efficiency and increasing HRT suggests that this reactor could reach discharge standards if left long enough; however, this would reduce the VTR and therefore increase costs.

The predicted energetic costs and forecasted tank size illustrate clearly the falling viability of MECs as COD concentration drops. In fact, with wastewaters with a COD below 600 mg/L (as would be typical of raw municipal or domestic wastewater [25]), the VTRs are extremely low, and HRTs would need to be very large to get organic removal down to the necessary level. However, with the high concentration wastewaters targeted in this pilot study, which are produced in all sites that de-water prior to anaerobic digestion, BES technology could be economically viable. The energetic cost savings versus AS could be huge when removing the first 50% of the COD load, and these savings may outweigh the large capital costs. It is acknowledged that performance would not stay the same when scaled up by such an order of magnitude, and maintenance costs have not been considered. However, the proposed OLR targets needed for economic viability [32] were met here, and elsewhere it has been shown that recycled carbon felt can effectively maintain or boost performance in a reactor design similar to this one, with a 88.6% reduction in cost [19].

Future pilot-scale research with this technology should seek to optimise performance, rather than demonstrate it. Improving CCE by optimising voltage input should help towards achieving energy neutrality. Improved understanding of, and ability to engineer biofilm formation processes could help improve CEs, generating more current. The exact nature of the biological processes and anaerobic metabolic pathways of BESs are unknown. They have been explored to an extent in laboratory studies [39,40,48] and can manifest themselves as strange observations when studies are carried out at scale, such as the disconnect between COD removal and current observed here. This in itself may not be a prohibiting factor in commercial uptake. The process of AS, discovered in 1913 [49], had revolutionised UK sanitation long before it was even known that the process was biological. However, the high variability within the reactor between identical commercially made electrode cassettes may be a greater challenge for industry to accept, as it introduces uncertainty and therefore risk. This variability has been documented in other pilot studies [1,2,10,50,51], and it shows that some electrodes are operating far below their potential. Major application of this technology cannot occur without further investigation into this electrode variability and analysis of the biofilm formation process. Finally, detailed understanding of how reactor components degrade over time will enable practical implementation at a larger scale.

This study is the first to rigorously optimise the HRT of a pilot-scale MEC treating real wastewater. VTRs were higher than ever previously reported, despite the fact that discharge standards were not reached. This was due to the use of RSL with a high organic content, rather than the use of raw wastewater. This study gives reason to view MECs differently. Instead of replacements for AS, further studies could look at using MECs as a pre-treatment technique for RSL to eliminate some of the AS cost. Theoretical calculations of the energetic cost savings indicates that the use of an MEC as a RSL treatment technique could result in an industrially viable system, despite the large capital costs. The use of a BES at this point in the treatment stream would remove the need to reach the legal discharge standards and reduce the need for large and high-risk industrial change. Using BES reactors here first could be an affordable transition phase that would help to improve the resilience, efficiency and reach of this technology.

## 4. Materials and Methods 

### 4.1. Reactor Configuration

**Figure 6:**
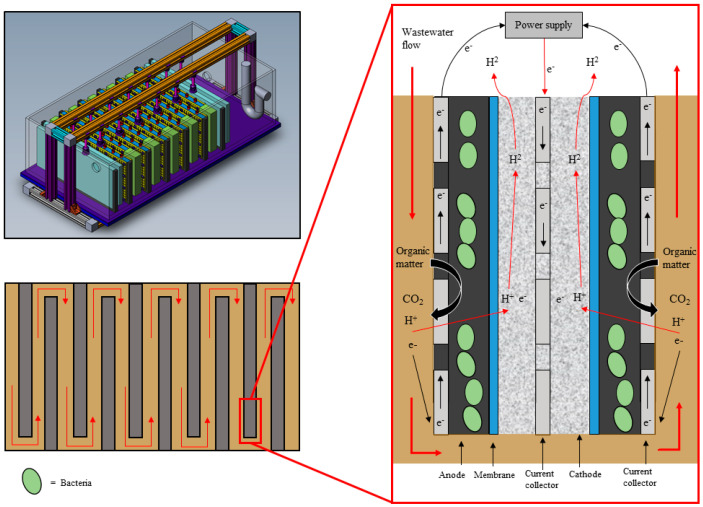
The pilot-scale MEC, showing the electrodes in the ‘cassette’ style design.

The reactor was built in the cassette design outlined by Heidrich et al. [10], with some modifications and improvements by BNC Solutions Ltd, a local engineering consultancy specialising in proof of concept development and new product design. It consisted of 10 cassettes set in a 72 L glass tank (Figure 6). Electrodes were made using an internal cathodic chamber filled with 10 g of stainless-steel wire, with stainless-steel mesh as a current collector (1 mm diameter, 10 mm × 10 mm openings). A wire was attached to the current collector using a steel clamp and fed through a sample port in the top of the reactor to connect to the power supply. Rhinohide membrane (Entec ©, Harrogate, UK) was used as a divider on either side of the cathode chamber. Following this, another plastic frame was used to seal the membrane and provide a frame for the carbon-felt anodes. On top of each of the anodes, a stainless-steel mesh was added as the external current collector. Again, a wire was attached to the current collector to connect to the power supply. Anodic working volume of the tank, when containing all ten cassettes, was 36 L. Anode surface area for each electrode was 0.0728 m^2^, giving the reactor a surface area to volume ratio of 20.2 m^2^/m^3^. Each cathodic compartment was filled with a phosphate buffer at a 0.5 M concentration. This was not replenished after inoculation. The cathodic compartments were sealed using a gasbag (Supel™ Inert Foil 1 L SCV Gas Sampling Bag with Thermogreen^®^ LB-2 Septa, Merck, Gillingham, UK) attached using Saint-Gobain Tygon Flexible Tubing (Tygon S3™ E-3603, RS, Corby, UK), at 6.4 mm external diameter.

Electrodes were placed in the tank to create a serpentine flow (Figure 6). Computational Fluid Dynamics (CFD) modelling on the tank was performed by the contracted engineering company to identify the optimal cassette spacing of 5 cm, and a false wall at the start of the flow was added to create laminar flow. Additionally, modelling showed that as the electrodes did not span the full width of the tank, PVC blocks needed to be added to the end of each cassette to provide a narrow gap for the water to travel around. The blocks were set on the outside of each cassette, minimising the ineffective areas at the corners as the RSL flowed around the electrodes.

### 4.2. Experimental Site and Operational Conditions

The reactor was situated at Northumbrian Water Ltd.’s wastewater treatment site at Howdon, South Banks, in their pump control building. Wastewater was piped up from the RSL pipe into a 1 m^3^ intermediate bulk container (IBC) which was filled and replenished every 2 days with fresh wastewater. The room was not heated, so the RSL remained at ambient temperature. 

The reactor was run from June 2019 to March 2020, with full access to the site. However, due to global events in March 2020, we were suddenly locked out of this site and we have been unable to retrieve this reactor or perform any further experiments. This prevented us from doing pertinent post analysis work in our laboratories that may have been destructive during the reactor’s operation. These include potentiostatic measurements, deconstruction of the reactor and taking biofilm samples. 

### 4.3. Analytical Methods 

COD was measured using Merck COD cuvette tests (500–10,000 mg/L). Soluble COD was measured as previously stated but using a 0.45 µm-filtered sample. Phosphate was measured using Merck Phosphate cuvette tests (0.05–5 mg/L and 3–100 mg/L). Sulphate was measured using Merck Sulphate cuvette tests (5–250 mg/L). Ammonia was measured using Merck cuvette tests (0.2–8 mg/L and 2–75 mg/L) using a 0.45 µm-filtered sample. Nitrite and Nitrate was measured using Ion Chromatograph (IC) Dionex ICS-1000. All samples were measured in duplicate. VFAs were measured using Ion Chromatography System Dionex Aquinion and AS-AP auto sampler. Filtered (0.2 µm PES syringe) samples were mixed with 0.1 M Octane Sulphonic Acid. Samples were then sonicated before analysis. All VFA samples were measured in triplicate.

Samples of the influent and effluent COD were measured three times a week during continuous flow mode. Voltage was measured over a 1-ohm resistor using Pico 6 software, with ADC-20 and ADC-24 PicoLogers for continuous measurement. The mean current density of all 10 electrodes was used for a total reactor current density. Hydrogen was measured in triplicates against an 80% hydrogen standard using a packed column in a Thermo Trace GC with a TCD, Argon carrier.4.4. 

### 4.4. Inoculation and Batch Mode 

The reactor was operated as an MEC for the entirety of the experiment, including start-up and acclimatisation. The inoculation of the reactor was done using 50% fresh RSL, 25% acetate mix (containing acetate, trace minerals and trace vitamins), and 25% effluent from lab-scale experimental work using MFCs, making up 36 L and filling the reactor. These MFCs were run using the same RSL mixed with a 0.25 g/L acetate concentration. The COD for this combined inoculation mix was 2656 mg/L. Initially, the reactor was run with the inoculum using a 16-day HRT, which, due to such a low flow rate, was operated in batch. This long HRT was used to increase reactor start-up speed [2] and to enhance biofilm development [46]. The reactor was then replenished with fresh RSL for three different retention times of 11 days, 9 days, and 2 days, with no further acetate supplementation.

### 4.5. Experimental Conditions

Each electrode was connected in series to a power supply, with 0.9 V added. Initially, once switched to continuous flow, the HRT was set to 0.25 days. This was doubled to 0.5 days and run for a further two weeks. Following this, the reactor was operated using the different HRTs of 0.015 days, 0.1 days, 0.25 days, 0.5 days, 1 day, 2 days, 6 days, and 18 days. These cycles were performed in a randomised order to eliminate the effect of time and biofilm maturation. 

### 4.6. Calculations

Voltage was measured using high resolution multi-channel data loggers from PicoTech [52]. This was converted to current density based on projected anode surface area. According to Ohms law, the current can be calculated as:

$$I=\frac{V}{R}$$

where I is the current (amps), V is the voltage (volts) and R is the resistance (Ohms). This was then divided by the surface area to get current density:

$$\frac{I}{A}=J$$

where A is the projected anode surface area (m^2^) and J is the current density (A/m^2^). 

CE was calculated by:

$$E_{c}=\frac{C_{p}}{C_{n}}\times 100$$

where E_c_ is the CE, C_p_ is the total coulombs (current over time), and C_n_ is the theoretical coulombs that could be recovered from the COD removed. Theoretical coulombs were calculated based on Logan et al [53]:

$$E_{c}=\frac{8\times C_{p}}{F\times v_{\mathrm{An}}\times ∆\mathrm{COD}\times T}\times 100$$

where F is Faraday’s constant (96,485 C/mol of electrons), 8 is a constant used for COD [53], v_An_ is the liquid volume in the anode chamber (L), ΔCOD is the change in COD (g/L) and T is the number of days for the measurement.

CCE was calculated by:

$$C_{c}=\frac{N_{H2}}{\left(C_{p}/\left(F\times 2\right)\right)}\times 100$$

where C_c_ is the CCE, N_H2_ the captured number of moles of H_2_, Cp is the total coulombs (current over time), F is Faraday’s constant and ‘2’ is to give moles of H_2_. 

VTR has been calculated using the COD removed and the retention time of the RSL, where S is COD removed (mg/L), HRT is the retention time (days), and Ks is VTR (kgCOD/m^3^∙day).

$$\frac{S}{\mathrm{HRT}\times 1000}=\mathrm{Ks}$$

The energetic treatment cost (kJ/gCOD) was calculated by taking the average watts and converting this to kJ per day, then dividing by the removal rate (gCOD/day).

$$\frac{W\times 86.4}{\mathrm{gCOD}\;\mathrm{per}\;\mathrm{day}}=\mathrm{KJ}/\mathrm{gCODr}$$

Hydrogen production was measured in litres of hydrogen per litre reactor per day. The total hydrogen produced from the whole reactor per week was divided by the reactor volume and then divided by the number of days over which it was collected.

$$\frac{{\mathrm{Total}\;H}_{2}\;\left(m^{3}\right)}{\left(\mathrm{Days}\times V\;\left(m^{3}\right)\right)}=\frac{m^{3}H_{2}}{m^{3}\cdot \mathrm{day}}$$

Energy recovered from hydrogen was based on a hydrogen volumetric density of 0.08988 kg/m^3^ and an energy density of 142 MJ/kg, and was reported as kJ/gCOD.

$$\frac{{\mathrm{Total}\;H}_{2}\left(m^{3}\right)}{\mathrm{Days}}\times \frac{\mathrm{Days}}{\mathrm{kgCODr}}\times 0.08988\times 142=\mathrm{KJ}/\mathrm{gCOD}$$

The equivalent cost for AS was calculated by the kg of COD removed per day, multiplied by the energetic treatment cost for AS (2 kJ/gCOD).

$$\frac{\mathrm{gCODr}}{\mathrm{day}}\times 2=\mathrm{AS}\;\mathrm{KJ}/\mathrm{day}$$

### 4.7. Tank Design

The actual flow rate for the RSL at Howdon Wastewater Treatment Site (NWL) is on average 100 L/s, equivalent to 8640 m^3^ per day. At a HRT of 0.5-days, a capacity of 4320 m^3^ is needed for the wastewater for one day of flow. However, as the electrode cassettes used in this study took up around 50% of the total tank, the theoretical tank size needed is 8640 m^3^. Based on a nominal depth of 3 m, which is similar dimensions to an AS lane [54,55], and a suggested width of 36 m, the VTR can then be modelled along the length of the tank, with 1 full day of flow requiring 80 m length. The energetic cost savings between each measured COD have been calculated using the experimental energetic costs calculated and the assumed energetic treatment cost of 2 kJ/gCOD removed for AS. This has then been converted to actual cost saved using an energy price of £3.6 ×10^−5^ /kJ [28,29].

## Figures and Tables

**Table 1:** Full wastewater components measured on a spot sample of return sludge liquor (RSL) taken prior to running the reactors on site.

COD mg/L	sCOD mg/L	Phosphate mg/L	Sulphate mg/L	Ammonia mg/L	Nitrate mg/L	Nitrite mg/L	Acetate mg/L	Butyric mg/L	Formic mg/L
4535	1772	56.8	180.6	298.3	8.9	0.8	274.0	49.2	n/a

**Table 2:** Average performance indicators for each hydraulic retention time (HRT). For hydrogen production values, averages have been taken from HRTs prior to core settling. HRTs measured after core settling have had their hydrogen production removed and are denoted by a hyphen (-).

HRT (Days)	Flow Rate (L/h)	Volumetric Treatment Rate (kgCOD/m^3^∙day)	COD Efficiency (%)	Current Density (A/m^2^)	Volumetric Hydrogen Production (m^3^/m^3^∙day)	Energetic Treatment Cost (kJ/gCOD)	Coulombic Efficiency (%)	Cathodic Conversion Efficiency (%)
0.015	100	0	0	1.36 ± 0.01	0	n/a	0	0
0.1	15	1.61 ± 2.27	9.3 ± 13.1	1.31 ± 0.07	0.0123 ± 0.0084	0.31 ± 0.44	2.9 ± 4.0	1.17 ± 0.86
0.25	6	3.09 ± 0.67	24.5 ± 1.6	1.21 ± 0.11	0.0155 ± 0.0076	0.64 ± 0.19	5.9 ± 1.8	1.16 ± 0.93
0.5	3	3.82 ± 1.94	52.4 ± 14.2	1.11 ± 0.08	0.0111 ± 0.0029	0.59 ± 0.42	5.6 ± 3.9	1.22 ± 0.25
1	1.5	1.81 ± 0.76	58.5 ± 7.4	1.10 ± 0.14	-	1.10 ± 0.46	9.9 ± 3.9	-
2	0.75	0.62 ±0.19	54.5 ± 2.3	1.17 ± 0.09	-	3.11 ± 0.73	28.0 ± 6.7	-
6	0.25	0.36 ± 0.14	76.2 ± 7.4	1.08 ± 0.04	-	5.25 ± 2.08	51.4 ± 20.5	-
18	0.083	0.13 ± 0.04	83.0 ± 12.9	1.12 ± 0.09	-	14.20 ± 5.04	131.0 ± 47.0	-

**Table 3:** Average VTRs from different influent CODs when subject to a flow rate of 3 L/h.

Influent COD (mg/L)	Volumetric Treatment Rate (kgCOD/m^3^∙day)	Time of Testing	Energetic Treatment Cost (kJ/gCOD)
4320	5.55	HRT optimisation	0.285
4153	5.27	HRT optimisation	0.36
3035	3.0	HRT optimisation	0.584
2376	1.43	Tank re-circulation	1.20
2186	1.47	HRT optimisation	1.13
950	0.282	Tank re-circulation	6.22
668	0.0985	Tank re-circulation	17.8
569	0.0653	Tank re-circulation	64.2
373	0.0275	Tank re-circulation	79.1
346	0.022	Tank re-circulation	52.1
324	0.0335	Tank re-circulation	94.1
290	0.0185	Tank re-circulation	79.1

## Supplementary Materials

The following are available online, Figure S1: The average current densities compared to HRT for the 10 cassette style electrodes.

## References

[1] Cusick R.D., Bryan B., Parker D.S., Merrill M.D., Mehanna M., Kiely P.D., Liu G., Logan B.E. (2011). Performance of a pilot-scale continuous flow microbial electrolysis cell fed winery wastewater. Appl. Microbiol. Biotechnol..

[2] Cotterill S.E., Dolfing J., Jones C., Curtis T.P., Heidrich E.S. (2017). Low Temperature Domestic Wastewater Treatment in a Microbial Electrolysis Cell with 1 m ^2^ Anodes: Towards System Scale-Up. Fuel Cells.

[3] Rousseau R., Etcheverry L., Roubaud E., Basséguy R., Délia M.L., Bergel A. (2020). Microbial electrolysis cell (MEC): Strengths, weaknesses and research needs from electrochemical engineering standpoint. Appl. Energy.

[4] Gajda I., Greenman J., Ieropoulos I.A. (2018). Recent advancements in real-world microbial fuel cell applications. Curr. Opin. Electrochem..

[5] Wang Z., He Z. (2020). Demystifying terms for understanding bioelectrochemical systems towards sustainable wastewater treatment. Curr. Opin. Electrochem..

[6] San-Martín M.I., Leicester D.D., Heidrich E.S., Alonso R.M., Mateos R., Escapa A. (2018). Bioelectrochemical Systems for Energy Valorization of Waste Streams. Energy Systems and Environment.

[7] Escapa A., Mateos R., Martínez E.J., Blanes J. (2016). Microbial electrolysis cells: An emerging technology for wastewater treatment and energy recovery. from laboratory to pilot plant and beyond. Renew. Sustain. Energy Rev..

[8] Rossi R., Jones D., Myung J., Zikmund E., Yang W., Gallego Y.A., Pant D., Evans P.J., Page M.A., Cropek D.M. (2019). Evaluating a multi-panel air cathode through electrochemical and biotic tests. Water Res..

[9] Cheng S., Liu W., Guo J., Sun D., Pan B., Ye Y., Ding W., Huang H., Li F. (2014). Effects of hydraulic pressure on the performance of single chamber air-cathode microbial fuel cells. Biosens. Bioelectron..

[10] Heidrich E.S., Dolfing J., Scott K., Edwards S.R., Jones C., Curtis T.P. (2013). Production of hydrogen from domestic wastewater in a pilot-scale microbial electrolysis cell. Appl. Microbiol. Biotechnol..

[11] Isabel San-Martín M., Mateos R., Carracedo B., Escapa A., Morán A. (2018). Pilot-scale bioelectrochemical system for simultaneous nitrogen and carbon removal in urban wastewater treatment plants. J. Biosci. Bioeng..

[12] San-Martín M.I., Sotres A., Alonso R.M., Díaz-Marcos J., Morán A., Escapa A. (2019). Assessing anodic microbial populations and membrane ageing in a pilot microbial electrolysis cell. Int. J. Hydrogen Energy.

[13] Baeza J.A., Martínez-Miró À., Guerrero J., Ruiz Y., Guisasola A. (2017). Bioelectrochemical hydrogen production from urban wastewater on a pilot scale. J. Power Sources.

[14] Brown R.K., Harnisch F., Wirth S., Wahlandt H., Dockhorn T., Dichtl N., Schröder U. (2014). Evaluating the effects of scaling up on the performance of bioelectrochemical systems using a technical scale microbial electrolysis cell. Bioresour. Technol..

[15] Escapa A., San-Martín M.I., Mateos R., Morán A. (2015). Scaling-up of membraneless microbial electrolysis cells (MECs) for domestic wastewater treatment: Bottlenecks and limitations. Bioresour. Technol..

[16] Gil-Carrera L., Escapa A., Mehta P., Santoyo G., Guiot S.R., Moran A., Tartakovsky B. (2013). Microbial electrolysis cell scale-up for combined wastewater treatment and hydrogen production. Bioresour Technol.

[17] Gil-Carrera L., Escapa A., Moreno R., Morán A. (2013). Reduced energy consumption during low strength domestic wastewater treatment in a semi-pilot tubular microbial electrolysis cell. J. Environ. Manag..

[18] Gil-Carrera L., Escapa A., Carracedo B., Morán A., Gómez X. (2013). Performance of a semi-pilot tubular microbial electrolysis cell (MEC) under several hydraulic retention times and applied voltages. Bioresour. Technol..

[19] Carlotta-Jones D.I., Purdy K., Kirwan K., Stratford J., Coles S.R. (2020). Improved hydrogen gas production in microbial electrolysis cells using inexpensive recycled carbon fibre fabrics. Bioresour. Technol..

[20] Ieropoulos I.A., Stinchcombe A., Gajda I., Forbes S., Merino-Jimenez I., Pasternak G., Sanchez-Herranz D., Greenman J. (2016). Pee power urinal-microbial fuel cell technology field trials in the context of sanitation. Environ. Sci. Water Res. Technol..

[21] Mills N., Pearce P., Farrow J., Thorpe R.B., Kirkby N.F. (2014). Environmental & economic life cycle assessment of current & future sewage sludge to energy technologies. Waste Manag..

[22] Department of Environment Food and Rural Affairs, UK (2011). Anaerobic Digestion Strategy and Action Plan. A Commitment to Increasing Energy from Waste through Anaerobic Digestion.

[23] Dai Z. (2019). Developing Energy and Nutrient Mass Balances to Inform Value Recovery Options in Municipal Wastewater Treatment Systems. Ph.D. Thesis.

[24] Dai Z., Heidrich E.S., Dolfing J., Jarvis A.P. (2019). Determination of the Relationship between the Energy Content of Municipal Wastewater and Its Chemical Oxygen Demand. Environ. Sci. Technol. Lett..

[25] Oakley M. (2018). Environmental engineering. Mil. Eng..

[26] Li W.W., Yu H.Q., He Z. (2014). Towards sustainable wastewater treatment by using microbial fuel cells-centered technologies. Energy Environ. Sci..

[27] Pant D., Singh A., Van Bogaert G., Gallego Y.A., Diels L., Vanbroekhoven K. (2011). An introduction to the life cycle assessment (LCA) of bioelectrochemical systems (BES) for sustainable energy and product generation: Relevance and key aspects. Renew. Sustain. Energy Rev..

[28] UKPower Compare Energy Prices Per kWh|Gas & Electric Per Unit|UKPower. https://www.ukpower.co.uk/home_energy/tariffs-per-unit-kwh.

[29] (2019). Business Electricity Prices Business Electricity Rates and Unit Prices per kWh. https://www.businesselectricityprices.org.uk/cost-per-kwh/.

[30] Carmona-Martínez A.A., Trably E., Milferstedt K., Lacroix R., Etcheverry L., Bernet N. (2015). Long-term continuous production of H2 in a microbial electrolysis cell (MEC) treating saline wastewater. Water Res..

[31] Kim K.Y., Yang W., Evans P.J., Logan B.E. (2016). Continuous treatment of high strength wastewaters using air-cathode microbial fuel cells. Bioresour. Technol..

[32] Aiken D.C., Curtis T.P., Heidrich E.S. (2019). Avenues to the financial viability of microbial electrolysis cells [MEC] for domestic wastewater treatment and hydrogen production. Int. J. Hydrogen Energy.

[33] Akman D., Cirik K., Ozdemir S., Ozkaya B., Cinar O. (2013). Bioelectricity generation in continuously-fed microbial fuel cell: Effects of anode electrode material and hydraulic retention time. Bioresour. Technol..

[34] Heidrich E.S., Edwards S.R., Dolfing J., Cotterill S.E., Curtis T.P. (2014). Performance of a pilot scale microbial electrolysis cell fed on domestic wastewater at ambient temperatures for a 12month period. Bioresour. Technol..

[35] Luo S., Jain A., Aguilera A., He Z. (2017). Effective control of biohythane composition through operational strategies in an innovative microbial electrolysis cell. Appl. Energy.

[36] Jiang D., Curtis M., Troop E., Scheible K., McGrath J., Hu B., Suib S., Raymond D., Li B. (2011). A pilot-scale study on utilizing multi-anode/cathode microbial fuel cells (MAC MFCs) to enhance the power production in wastewater treatment. Int. J. Hydrogen Energy.

[37] Yildiz B.S. (2012). Water and wastewater treatment: Biological processes. Metropolitan Sustainability: Understanding and Improving the Urban Environment.

[38] Recio-Garrido D., Perrier M., Tartakovsky B. (2016). Modeling, optimization and control of bioelectrochemical systems. Chem. Eng. J..

[39] Zhao F., Heidrich E.S., Curtis T.P., Dolfing J. (2020). The effect of anode potential on current production from complex substrates in bioelectrochemical systems: A case study with glucose. Appl. Microbiol. Biotechnol..

[40] Zhao F., Heidrich E.S., Curtis T.P., Dolfing J. (2020). Understanding the complexity of wastewater: The combined impacts of carbohydrates and sulphate on the performance of bioelectrochemical systems. Water Res..

[41] Ma J., Wang Z., He D., Li Y., Wu Z. (2015). Long-term investigation of a novel electrochemical membrane bioreactor for low-strength municipal wastewater treatment. Water Res..

[42] Jadhav D.A., Ghangrekar M.M., Duteanu N. (2017). Recent progress towards scaling up of MFCs. Microbial Fuel Cell: A Bioelectrochemical System that Converts Waste to Watts.

[43] Sander R. (2015). Compilation of Henry’s law constants (version 4.0) for water as solvent. Atmos. Chem. Phys..

[44] Ali S.F. (2019). Determining the UK’s potential for heat recovery from wastewater using steady state and dynamic modelling-preliminary results. WEENTECH Proc. Energy.

[45] Emmanuel Alepu O., Li Z. (2016). Effect of Hydraulic Retention Time on Anaerobic Digestion of Xiao Jiahe Municipal Sludge. Int. J. Waste Resour..

[46] Ichihashi O., Vishnivetskaya T.A., Borole A.P. (2014). High-Performance Bioanode Development for Fermentable Substrates via Controlled Electroactive Biofilm Growth. ChemElectroChem.

[47] Council of European Communities (CEC) (1991). EU Council Directive of 21 May 1991 concerning urban waste water treatment (91/271/EEC). Off. J. Eur. Communities.

[48] Velasquez-Orta S.B., Yu E., Katuri K.P., Head I.M., Curtis T.P., Scott K. (2011). Evaluation of hydrolysis and fermentation rates in microbial fuel cells. Appl. Microbiol. Biotechnol..

[49] Wiesmann U., Choi I.S., Dombrowski E.-M. (2006). Historical Development of Wastewater Collection and Treatment. Fundamentals of Biological Wastewater Treatment.

[50] Ge Z., He Z. (2016). Long-term performance of a 200 L modularized microbial fuel cell system treating municipal wastewater: Treatment, energy, and cost. Environ. Sci. Water Res. Technol..

[51] Linares R.V., Domínguez-Maldonado J., Rodríguez-Leal E., Patrón G., Castillo-Hernández A., Miranda A., Romero D.D., Moreno-Cervera R., Camara-chale G., Borroto C.G. (2019). Scale up of microbial fuel cell stack system for residential wastewater treatment in continuous mode operation. Water.

[52] PicoTech High-Resolution Data Acquisition|Pico Technology. https://www.picotech.com/data-logger/adc-20-adc-24/precision-data-acquisition.

[53] Logan B.E. (2008). Microbial Fuel Cells.

[54] Eckenfelder W. (1998). Activated Sludge: Process Design and Control.

[55] Japan Sewage Works Association Design Standard for Municipal Wastewater Treatment Plants. gcus.jp/wp/wp-content/.../735735ded23d4d28db9cc4f879e8da24.pdf.

