# Supplementary material for: Optimising the Hydraulic Retention Time in a Pilot-Scale Microbial Electrolysis Cell to Achieve High Volumetric Treatment Rates Using Concentrated Domestic Wastewater
Source: Molecules. 2020 Jun 26;25(12):2945. doi: 10.3390/molecules25122945 (PMC7356006; doi:10.3390/molecules25122945)
Supplement: Supplementary file 1 [file molecules-25-02945-s001.pdf]

Supplementary information for

Optimising the Hydraulic Retention Time in a Pilot-Scale Microbial Electrolysis Cell to Achieve High Volumetric Treatment Rates Using Concentrated Domestic Wastewater

Daniel D. Leicester [1], Jaime M. Amezaga [1], Andrew Moore [2] and Elizabeth S. Heidrich [1,*]

[1] School of Engineering, Newcastle University, Newcastle-upon-Tyne NE1 7RU, UK
[2] Northumbrian Water Limited, Northmbria House, Abbey Road, Durham DH1 5FJ, UK

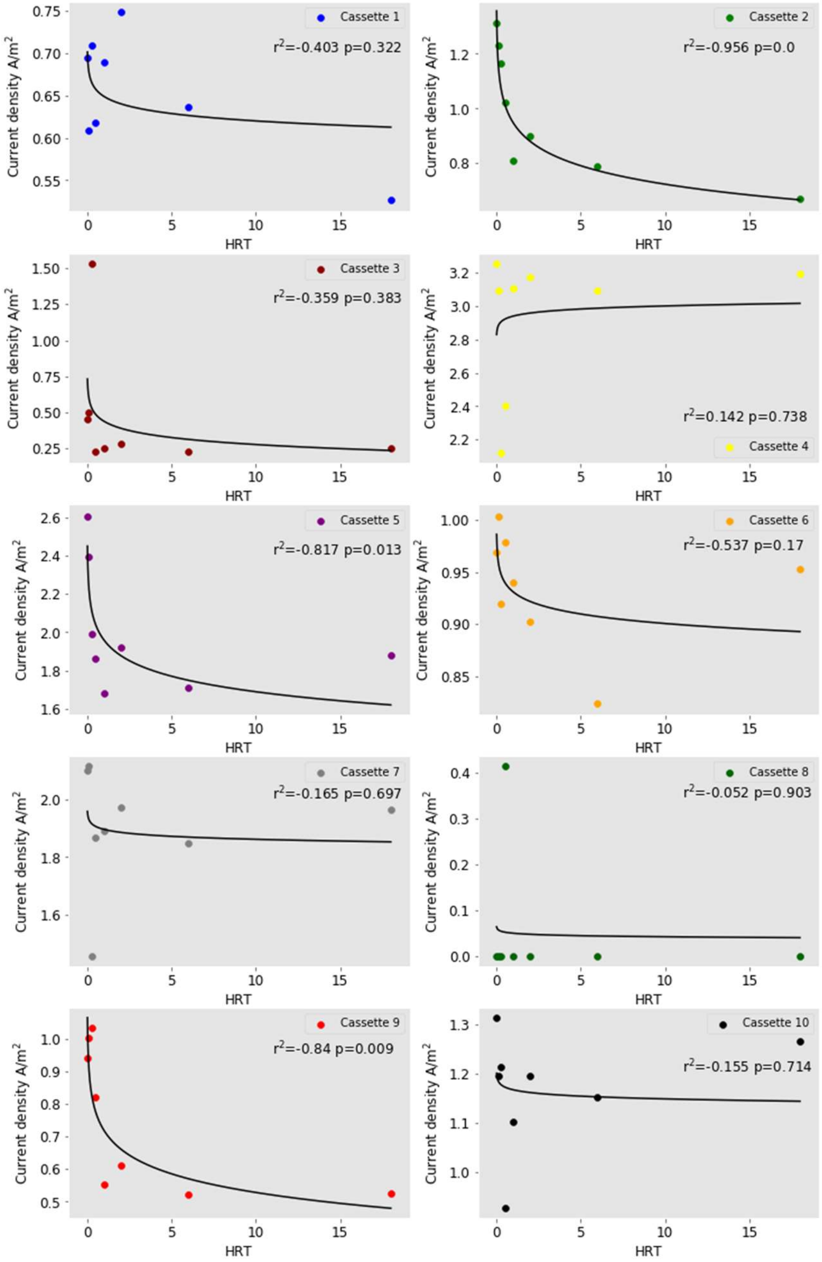

Figure S1. The average current densities compared to HRT for the 10-cassette style electrodes.
